# Calibrated term presence modelling for CCUS patent abstracts: a case study with variational autoencoders and retrieval baselines

**DOI:** 10.1098/rsos.261365

**Published:** 2026-10-28

**Authors:** Charalampos Matsantonis, George Tzougas, Dimitris Christopoulos, David Dekker, George Streftaris

**Affiliations:** ^1^ Mathematics-Driven Innovation Centre and The Maxwell Institute for Mathematical Sciences, Heriot-Watt University, Edinburgh, UK; ^2^ School of Mathematical and Computer Sciences and The Maxwell Institute for Mathematical Sciences, Duncan Laboratory for Insurance Data Science, Heriot-Watt University, Edinburgh, UK; ^3^ Edinburgh Business School, Heriot-Watt University, Edinburgh, UK

**Keywords:** patent text mining, carbon capture, utilization and storage, cooperative patent classification, probability calibration, variational autoencoder, patent retrieval

## Abstract

Patent codes may lack granularity when carbon capture, utilization and storage (CCUS) inventions combine climate and chemical engineering concepts. We evaluate term presence models on 10 603 English patent abstracts through document completion, calibration and cooperative patent classification (CPC) prefix retrieval. We compare two variational autoencoders (VAEs), a product of the experts latent Dirichlet allocation model, a unigram prior, term frequency-inverse document frequency (TF-IDF), PatentSBERTa and PaECTER. With a target masking rate of 50 per cent for positive terms, TF-IDF nearest neighbours give the best ranked completion. The direct Bernoulli VAE ranks slightly above the negative binomial to Bernoulli VAE (NB VAE), while the latter has lower expected calibration error (ECE). Conditional posterior predictive draws retain positive rank associations for document and term counts, but the NB VAE and direct Bernoulli VAE predict 1.47 and 1.63 times the observed positive count, respectively. PaECTER and the TF-IDF representation after singular value decomposition (SVD) give the strongest CPC prefix retrieval, while VAE probabilities could support inspectable term screening.

## 1. Introduction

Patent data are widely used to study innovation, firm performance and technological change because each document combines technical text, classification codes, citations and bibliographic metadata [1–3]. In climate and energy research, these data are particularly important because they provide a detailed record of inventions related to carbon capture, utilization and storage (CCUS) and other mitigation technologies [4,5]. However, the value of patent evidence depends on whether the relevant technological domain can be identified at the level of detail required by the question being asked.

Cooperative patent classification (CPC) is the classification system used throughout this paper. Within CPC, section Y covers general tagging of new technological developments and technologies that span several fields [6–8]. Class Y02 covers technologies or applications for mitigation or adaptation against climate change, and subclass Y02C covers capture, storage, sequestration or disposal of greenhouse gases [6,9]. Thus, Y02 is not a separate taxonomy outside CPC, it is a CPC class, and Y02C is the greenhouse gas subclass that is especially relevant for CCUS. Because the CPC system also includes technical codes in chemical engineering, particularly gas separation and purification codes under B01D, this study combines CPC symbols related to climate and industrial processes [6,7,10].

This terminology matters because the problem addressed here is not that CPC labels are uninformative. On the contrary, CPC labels provide a structured reference that is useful for search and technology monitoring [6–9]. However, a view based on codes can be incomplete when the objective is to separate technical mechanisms inside a broad climate area. For example, a search that begins with Y02C may still need to distinguish direct air capture sorbents, membrane-based capture, amine regeneration and pressure swing adsorption [5], while also identifying documents that combine climate tags with chemical engineering vocabulary. The European Patent Office explains that inventories of Y codes are updated through search algorithms designed by experts and relying on other CPC symbols, International Patent Classification symbols and keywords [11]. Text evidence is therefore part of the update process, while the resulting tags do not provide calibrated probabilities for individual documents.

Recent patent text research provides several ways to construct evidence for individual documents. Transformer models trained for patents have been used for classification and similarity search [12–14], building on general transformer language and sentence representation architectures [15,16]. PatentSBERTa, an augmented sentence transformer model, and patent embeddings using citation informed TransformERs (PaECTER) provide patent representations for semantic retrieval [13,14]. Variational autoencoders (VAEs) provide a general framework for latent variable generative modelling [17]. Finally, neural topic models and text specific VAE variants have been developed for document representation, topic modelling, document completion and sparse discrete data [18–21]. These methods are commonly evaluated through classification accuracy, similarity ranking, perplexity or topic coherence. Calibrated term presence modelling asks a different question. Given a partially observed abstract, how probable is it that each vocabulary term appears in that document?

This task occupies a useful place between administrative classification and similarity based on embeddings. While a code label states that a document belongs to a classification category and an embedding provides a geometric neighbourhood, a calibrated term presence model instead gives auditable probabilities for each term that can be thresholded, ranked, compared across documents and checked against empirical frequencies.

The paper is therefore a CCUS case study rather than a general methodological advance. The empirical design uses training, validation and test shares of 80 per cent, 10 per cent and 10 per cent, respectively. It combines a fixed document completion protocol, calibration diagnostics, a conditional posterior predictive sparsity assessment and a downstream CPC prefix retrieval task for gas separation labels inside the filtered corpus. The negative binomial to Bernoulli VAE (NB VAE) is compared with a direct Bernoulli VAE, the product of the experts latent Dirichlet allocation model (ProdLDA), a unigram prior, term frequency-inverse document frequency (TF-IDF) nearest neighbour completion, PatentSBERTa nearest neighbour completion and PaECTER nearest neighbour completion. For CPC prefix retrieval, VAE latent means, ProdLDA topic proportions, TF-IDF after singular value decomposition (SVD) and PaECTER embeddings are evaluated under the same logistic regression specification.

The contribution is empirical and deliberately focused. The NB VAE provides low aggregate calibration errors and a compact latent representation with CPC retrieval signal. However, conditional posterior predictive draws overstate the number of positive terms among unobserved coordinates. Low aggregate calibration error should therefore not be read as complete reproduction of document sparsity. The results also show that this VAE is not the strongest ranked retrieval method. TF-IDF nearest neighbours recover held out positive terms better at the top of the list, while PaECTER or TF-IDF representations are stronger for CPC prefix retrieval. This complementary interpretation supports term presence modelling as an additional evidence layer for CCUS patent analysis and not as a replacement for CPC labels, lexical search or pretrained patent embeddings.

The paper is structured as follows: corpus construction is described in §2.1 and the study tasks in §2.2. The NB VAE is described in §3.1, the baselines in §3.2, the shared metrics in §3.3 and the conditional posterior predictive design in §3.4. We report model selection in §4.1, document completion in §4.2, calibration in §4.3, conditional sparsity reproduction in §4.4 and CPC prefix retrieval in §4.5. In §5, we discuss the combined evidence and its limitations. In Appendices A–C we provide derivations, reproducibility details and the full conditional posterior predictive results.

## 2. Corpus, representation and study design

### 2.1. Corpus construction and text representation

The empirical corpus is constructed from English language patent records with abstracts and CPC metadata. Let $d_{i}$ denote patent document i and let $C_{i}$ be the set of normalized CPC symbols assigned to that document. We include a document if at least one CPC symbol begins with a prefix in the CCUS related prefix set. This set is denoted by P and is defined as:

(2.1) P={Y02C,B01D53/04,B01D53/46,B01D53/62}.

The relation c⪰p means that the CPC symbol c begins with prefix p. The filtered CCUS corpus is therefore given by:

(2.2) $$D_{\mathrm{CCUS}}=\{d_{i}|\exists c\in C_{i},\exists p\in P\;\mathrm{such}\;\mathrm{that}\;c⪰p\}.$$

For the binary term presence representation, the count vectorizer converts each abstract to lower case, treats punctuation as token boundaries, applies its English stopword list and retains alphabetic strings of at least two characters using the letters a to z. We do not use stemming or lemmatization because patent abstracts often contain technical distinctions where surface forms are meaningful, such as adsorption, adsorbent and adsorbing. This preprocessing favours transparent analysis of the vocabulary over chemical entity normalization. Consequently, chemical formulae and alphanumeric identifiers are not preserved. This is a limitation for patent mining in chemistry, but it keeps the document representation reproducible and directly aligned with the term presence task.

Let $M_{\mathrm{train}}$ be the number of training documents and let ${\mathrm{df}}_{\mathrm{train}}(t)$ be the number of training documents in which term t appears. The retained vocabulary V is fitted on the training split alone and is defined by document frequency filtering:

(2.3) $$V=\{t|5\leq {\mathrm{df}}_{\mathrm{train}}(t)\leq 0.95M_{\mathrm{train}}\}.$$

The lower threshold removes low-frequency terms, while the upper threshold removes terms that are too common to carry information specific to a document in the training corpus, and the fitted vocabulary is then applied unchanged to the validation and test abstracts. Let |V| denote the vocabulary size, let $M_{\mathrm{val}}$ and $M_{\mathrm{test}}$ denote the validation and test document counts and let $M=M_{\mathrm{train}}+M_{\mathrm{val}}+M_{\mathrm{test}}$ denote the total document count. Each document is mapped to a binary vector $x_{i}\in \{0,1{\}}^{\left|V\right|}$, whose entry $x_{iv}$ is one when term v appears at least once in document i and zero otherwise. Stacking all vectors gives the document term matrix $X\in \{0,1{\}}^{M\times |V|}$. Table 1 lists the resulting corpus statistics.

**Table 1.** Corpus statistics after CPC filtering, text preprocessing and vocabulary construction using the training split only. The training, validation and test shares are 80 per cent, 10 per cent and 10 per cent, respectively. This division separates model selection from final reporting.

quantity	symbol	value
documents	M	10 603
training documents	$M_{\mathrm{train}}$	8481
validation documents	$M_{\mathrm{val}}$	1061
test documents	$M_{\mathrm{test}}$	1061
vocabulary size	|V|	4412

Using abstracts and binary term presence is a restrictive design choice as it discards claims, descriptions, term counts, phrase boundaries and chemical notation. However, this restriction is appropriate for the present case study because the target output is the probability that a vocabulary term appears at least once in a short patent abstract. By keeping the representation simple, the evaluation can focus on calibration, document completion and comparison with stronger retrieval baselines rather than on the effects of full text extraction or domain specific chemical tokenization.

### 2.2. Evaluation tasks and reproducibility design

The first target task is document completion. For each validation or test abstract containing at least two positive terms from the retained vocabulary, a fixed random set is held out using a target masking rate of 50 per cent while at least one term remains revealed. Each model must recover the held out positive terms from the revealed positive terms. This protocol mirrors a setting with partial evidence in which observed vocabulary may imply related technical vocabulary. It also evaluates predictions on unobserved coordinates rather than rewarding a model for reconstructing terms already supplied as input.

The second target task is calibration on the same unobserved document term pairs. Calibration matters because the VAE output is intended to be interpreted as a probability signal, not only as a ranking score. Since almost all possible document term pairs are absent, calibration is evaluated with reliability diagrams, expected calibration error (ECE) and Brier score. These diagnostics are complemented by calibration stratified by training document frequency so that the abundant rare term pairs do not obscure behaviour for more common vocabulary.

The third component is an additional conditional posterior predictive sparsity assessment for the two fitted VAE variants. It samples complete binary outcomes over the unobserved coordinates while holding revealed positive terms fixed. It then compares positive counts for each document and document counts for each term among the unobserved coordinates. This is a model check rather than a model selection task. Its design is defined in §3.4.

The final target task is CPC prefix retrieval. Because the corpus is already filtered around Y02C and related separation codes, Y02C itself is not used as a binary target inside the filtered data. Instead, the downstream task evaluates retrieval for technical prefixes with sufficient positive and negative examples, namely B01D53/04 and B01D53/62. These labels are not treated as perfect ground truth for technological meaning. Instead, they provide an external administrative reference that tests whether a text representation can recover known gas separation assignments.

The corpus is divided with seed 10 into training, validation and test shares of 80 per cent, 10 per cent and 10 per cent, respectively. When every joint class contains enough observations, the split is stratified by the joint B01D53/04 and B01D53/62 label so that the downstream targets remain represented across partitions. All final performance numbers are reported on the test split reserved for final evaluation. Hyperparameters and early stopping are selected on the validation split, and the test split is not used for model selection. Appendix B records the deterministic settings, training schedule, mask construction, nearest neighbour configuration and downstream retrieval procedure.

## 3. Models and evaluation protocol

### 3.1. Negative binomial to Bernoulli variational autoencoder (NB VAE)

The case study model is a VAE for binary term presence and is referred to as the NB VAE. Let $R^{K}$ denote the real vector space with dimension K. Each document has a latent vector $z_{i}\in R^{K}$, where K is the latent dimension, 0 is the zero vector with K entries and $I_{K}$ is the K×K identity matrix. The prior over latent vectors is the standard multivariate normal distribution:

(3.1) $$p(z_{i})=N(0,I_{K}).$$

Let ϕ denote the encoder parameters, $\mu_{i}\in R^{K}$ denote the encoder mean and $\sigma_{i}\in (0,\infty {)}^{K}$ denote the vector of elementwise standard deviations, so $\sigma_{i}^{2}$ is its elementwise variance. The operator diag(⋅) places a vector on the diagonal of a matrix. The encoder maps $x_{i}$ to the variational distribution:

(3.2) $$q_{\phi }(z_{i}|x_{i})=N(\mu_{i},\mathrm{diag}(\sigma_{i}^{2})).$$

Let $\epsilon_{i}∼N(0,I_{K})$ be an auxiliary random vector and let ⊙ denote elementwise multiplication. During training, the reparametrization is:

(3.3) $$z_{i}=\mu_{i}+\sigma_{i}⊙\epsilon_{i}.$$

Let θ denote the decoder parameters. For document i and vocabulary coordinate v, the decoder produces a count logit $a_{iv}$ and a zero probability logit $b_{iv}$. Before defining their transformations, let $a_{\max}>0$ be a fixed clipping constant, let clip(u,l,r) truncate the real value u to the interval [l,r] and let σ(u)=1/(1+exp⁡(−u)) be the logistic sigmoid. The transformed decoder parameters are:

(3.4) $$\begin{array}{rl} \alpha_{iv} & =\exp\{\mathrm{clip}(a_{iv},-a_{\max},a_{\max})\}\\ \text{and }\rho_{iv} & =\sigma (-b_{iv}). \end{array}$$

A latent count $m_{iv}$ is introduced only to derive a Bernoulli presence probability. Let m∈{0,1,2,…} denote a possible count and let Γ(⋅) denote the gamma function. Conditional on $z_{i}$, the count probability mass function is:

(3.5) $$\mathrm{Pr}(m_{iv}=m|\alpha_{iv},\rho_{iv})=\frac{\Gamma (m+\alpha_{iv})}{\Gamma (\alpha_{iv})\Gamma (m+1)}(1-\rho_{iv}{)}^{m}\rho_{iv}^{\alpha_{iv}}.$$

Under this parametrization, the probability of a zero latent count is $\rho_{iv}^{\alpha_{iv}}$. The Bernoulli probability $\pi_{iv}$ that term v appears at least once in document i is therefore:

(3.6) $$\pi_{iv}=\mathrm{Pr}(m_{iv}\geq 1|z_{i})=1-\rho_{iv}^{\alpha_{iv}}.$$

The observed binary term presence variable is modelled as:

(3.7) $$x_{iv}|z_{i}∼\mathrm{Bernoulli}(\pi_{iv}).$$

Because the observed data are binary, the likelihood depends on the decoder only through $\pi_{iv}$. The quantities $\alpha_{iv}$ and $\rho_{iv}$ are decoder link parameters and are not separately interpreted as parameters of observed patent word counts. Appendix B records the numerical safeguards used to evaluate this link. Assuming conditional independence across vocabulary coordinates given $z_{i}$, the decoder likelihood is:

(3.8) $$p_{\theta }(x_{i}|z_{i})=\prod_{v=1}^{\left|V\right|}\pi_{iv}^{x_{iv}}(1-\pi_{iv}{)}^{1-x_{iv}}.$$

Let e denote the training epoch and $T_{\mathrm{warm}}$ denote the number of warm-up epochs. The warm-up coefficient is:

(3.9) $$\beta_{e}=\min\{1,e/T_{\mathrm{warm}}\}.$$

Let $D_{\mathrm{KL}}(q\|p)$ denote the Kullback-Leibler (KL) divergence from a distribution q to a distribution p, and let $E_{q}[\cdot ]$ denote expectation under q. The training objective at epoch e is denoted $J_{e}(x_{i})$ and is the negative reconstruction term plus the KL divergence weighted by the warm-up coefficient:

(3.10) $$J_{e}(x_{i})=-E_{q_{\phi }(z_{i}|x_{i})}[\log p_{\theta }(x_{i}|z_{i})]+\beta_{e}D_{\mathrm{KL}}(q_{\phi }(z_{i}|x_{i})\|p(z_{i})).$$

At $\beta_{e}=1$, this objective is the negative of the evidence lower bound (ELBO). Sections A.1–A.4 of appendix A give the negative binomial to Bernoulli derivation, the stable log likelihood, the ELBO and the diagonal Gaussian KL term.

### 3.2. Baselines and ablations

To isolate the components associated with performance, the NB VAE is compared with targeted baselines and ablations. A direct Bernoulli VAE uses the same encoder architecture but replaces the negative binomial mapping with a single sigmoid decoder. ProdLDA is a product of the experts neural topic model [19]. In this study, it is trained on the same binary bag-of-words representation. A unigram prior assigns each term its training set presence rate and therefore removes document conditioning entirely. All three neural models are trained on complete training vectors, and masking is applied only to validation and test inputs for model selection and evaluation.

Nearest neighbour baselines compare similarity-based retrieval with generative text modelling. TF-IDF and cosine similarity follow standard information retrieval definitions [22]. The TF-IDF baseline finds the 30 closest training abstracts using cosine similarity and forms a normalized non-negative average of their binary term presence vectors, with weights obtained by truncating cosine similarity at zero and adding a $10^{-6}$ offset before normalization. Two patent transformer nearest neighbour baselines are also evaluated. PatentSBERTa tests a sentence transformer representation developed for patent distance and classification, whereas PaECTER tests a patent encoder informed by citations and designed for semantic similarity between patents [13,14]. The training embeddings are computed from complete training abstracts, and each completion query is a constructed abstract containing only the revealed vocabulary terms separated by spaces. It contains at most 256 terms and does not retain the original word order. Consequently, held out positive terms cannot enter the query.

The downstream CPC prefix task uses the same retrieval model specification. VAE latent means, ProdLDA topic proportions, TF-IDF vectors after SVD and PaECTER embeddings are passed to logistic regression with balanced class weights. Full abstract representations are used for this downstream task. This design facilitates comparison of the representations under a common supervised model specification.

### 3.3. Shared completion, calibration and retrieval metrics

For a validation or test document i, define the positive term set as $S_{i}=\{v|x_{iv}=1\}$. For any finite set A, |A| denotes its cardinality, and ⌊u⌋ denotes the greatest integer not exceeding the real value u. If $|S_{i}|\geq 2$, a fixed random partition divides the positive terms into a revealed positive term set $O_{i}$ and a held out positive term set $H_{i}$ using masking rate η=0.5. The partition satisfies:

(3.11) $$\begin{array}{rl} O_{i}\cup H_{i} & =S_{i},\\ O_{i}\cap H_{i} & =\emptyset \\ \text{and }|H_{i}| & =\max\{1,\lfloor \eta |S_{i}|\rfloor \}. \end{array}$$

For a singleton $S_{i}$, its one positive term remains revealed and $H_{i}$ is empty. Completion and calibration summaries retain only documents with $|H_{i}|>0$. All 1061 test documents satisfy this eligibility condition.

The observed vector $x_{i}^{\mathrm{obs}}$ has entry $x_{iv}^{\mathrm{obs}}=1$ for $v\in O_{i}$ and zero otherwise. For completion and calibration, each VAE decoder is evaluated deterministically at the encoder mean $z_{i}=\mu_{i}$. Latent sampling is reserved for the conditional posterior predictive assessment in §3.4. For any statement G, let I{G} be one when G is true and zero otherwise. Candidate rankings use the original non-negative model scores $s_{iv}$, with revealed terms excluded. For the completion perplexity (PPL) calculation, let $\varepsilon_{s}=10^{-12}$ and define the stabilized candidate score:

(3.12) $${\bar{s}}_{iv}=\max\{s_{iv},\varepsilon_{s}\}I\{v\notin O_{i}\}.$$

Let u denote an index for a vocabulary coordinate used in the denominator. The normalized completion distribution is:

(3.13) $${\tilde{p}}_{iv}=\frac{{\bar{s}}_{iv}}{\sum_{u=1}^{\left|V\right|}{\bar{s}}_{iu}},$$

when the denominator is finite and positive. If the denominator is not finite or positive, the implementation uses a uniform distribution over the unobserved coordinates as a numerical safeguard. Let log and exp denote the natural logarithm and exponential functions. PPL is computed only on held out positive terms and pooled across eligible documents as:

(3.14) $$\mathrm{PPL}=\exp\left(-\frac{\sum_{i}\sum_{v\in H_{i}}\log{\tilde{p}}_{iv}}{\sum_{i}|H_{i}|}\right).$$

This quantity is a diagnostic for completing held out positive terms and not a perplexity derived from the likelihood of a language model.

For a positive integer cut-off k, precision at k is the fraction of the top k unobserved coordinates that belong to $H_{i}$, and recall at k is the fraction of $H_{i}$ recovered among those coordinates. Normalized discounted cumulative gain (nDCG) at k discounts a correct held out positive term by its logarithmic rank and divides by the ideal gain for that document. The abbreviations P@k, R@k and nDCG@k denote precision at k, recall at k and nDCG at k, respectively. Table 2 and figures 1 and 2 use these abbreviations. These metrics are averaged across eligible documents for k∈{5,10,20}. Recall and nDCG are also plotted for cut-offs from 1 to 50.

**Figure 1.**
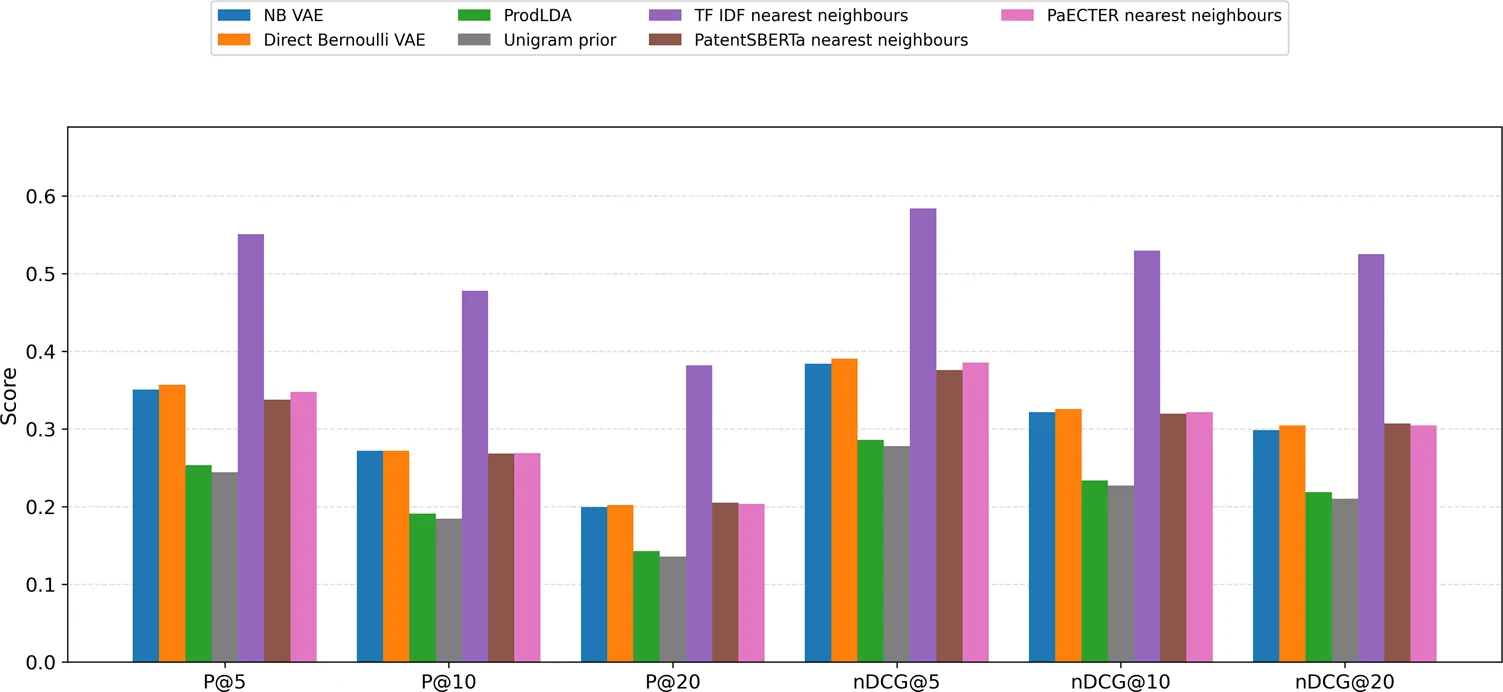
Top-ranked document completion favours TF-IDF nearest neighbours. The VAE variants remain close to the PatentSBERTa and PaECTER nearest neighbour baselines and are stronger than ProdLDA and the unigram prior on the displayed ranking metrics.

**Figure 2.**
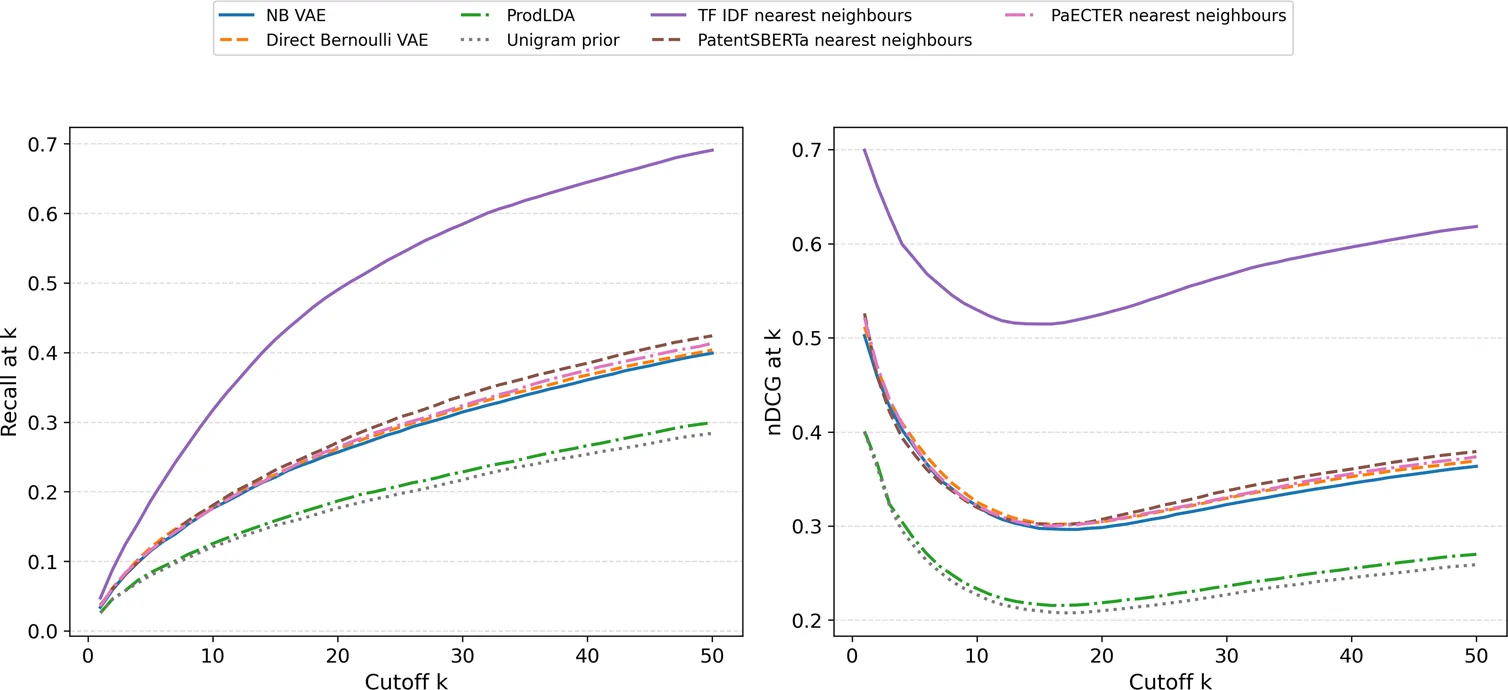
Rank curves show that TF-IDF nearest neighbours retain the highest recall and normalized discounted cumulative gain (nDCG) over cut-offs from 1 to 50, while the curves for the VAE variants remain above those for the unigram prior and ProdLDA.

**Table 2.** Document completion results on the test split reserved for final evaluation. Revealed positive terms are excluded from the candidate ranking. Higher values are better for P@5, R@20 and nDCG@5. Lower PPL is better. The best value in each column is shown in bold.

model	P@5	R@20	nDCG@5	PPL
NB VAE	0.351	0.257	0.384	578.908
direct Bernoulli VAE	0.357	0.262	0.391	**573.735**
ProdLDA	0.253	0.187	0.286	987.577
TF-IDF nearest neighbours	**0.551**	**0.490**	**0.584**	2695.356
PatentSBERTa nearest neighbours	0.338	0.271	0.376	10 176.732
PaECTER nearest neighbours	0.348	0.264	0.385	14 105.749
unigram prior	0.244	0.176	0.278	1101.841

Calibration is evaluated on the set of unobserved document term pairs:

(3.15) $$E=\{(i,v)||H_{i}|>0,\;v\notin O_{i}\}.$$

For each pair, the binary target is $y_{iv}=x_{iv}$ and the model prediction is denoted ${\hat{p}}_{iv}\in [0,1]$. The Brier score [23] is:

(3.16) $$\mathrm{Brier}=\frac{1}{\left|E\right|}\sum_{(i,v)\in E}({\hat{p}}_{iv}-y_{iv}{)}^{2}.$$

To compute ECE, [0,1] is divided into B=15 bins of equal width indexed by b∈{1,…,B}. Let $E_{b}\subseteq E$ contain the pairs whose predictions fall in bin b and define the set of non-empty bins:

(3.17) $$B_{+}=\{b\in \{1,\ldots ,B\}||E_{b}|>0\}.$$

For each $b\in B_{+}$, define the mean prediction as:

(3.18) $${\mathrm{conf}}_{b}=\frac{1}{\left|E_{b}\right|}\sum_{(i,v)\in E_{b}}{\hat{p}}_{iv},$$

and the empirical frequency is:

(3.19) $${\mathrm{freq}}_{b}=\frac{1}{\left|E_{b}\right|}\sum_{(i,v)\in E_{b}}y_{iv}.$$

The error weighted by bin size is:

(3.20) $$\mathrm{ECE}=\sum_{b\in B_{+}}\frac{\left|E_{b}\right|}{\left|E\right|}|{\mathrm{freq}}_{b}-{\mathrm{conf}}_{b}|.$$

The reliability diagram includes the NB VAE share of evaluation pairs in each bin because an ordinary diagram does not display the proportion of observations in each bin, whereas ECE weights each bin by that proportion [24,25]. This information is important for sparse term presence data because bins with higher probabilities can contain few observations. Calibration is also reported for rare, medium and common terms, defined by tertiles of training set document frequency.

For the downstream CPC prefix task, let g denote a target prefix. Its binary label for document i is:

(3.21) $$y_{i}^{\left(g\right)}=I\{\exists c\in C_{i}\;\mathrm{such}\;\mathrm{that}\;c⪰g\}.$$

The representations are VAE latent means, ProdLDA topic proportions, TF-IDF representations reduced by SVD [26] and PaECTER embeddings. A logistic regression model is fitted on the training split and evaluated on the test split. We report the area under the receiver operating characteristic curve (AUROC), average precision (AP) and precision among the top 100 ranked test patents (P@100). AP is primary because the positive label is imbalanced within the filtered CCUS corpus.

### 3.4. Conditional posterior predictive sparsity assessment

The two fitted VAE variants are additionally assessed on the same fixed test inputs and mask used for completion. Let $I=\{i||H_{i}|>0\}$ be the eligible test document set. For document i∈I, define the unobserved coordinate set $U_{i}=V\setminus O_{i}$. Let R=1000 be the number of Monte Carlo draws, and let r∈{1,…,R} index a draw. For each model and draw, a latent vector is sampled from the fitted encoder as follows:

(3.22) $$z_{i}^{\left(r\right)}∼q_{\phi }(z_{i}|x_{i}^{\mathrm{obs}}).$$

In this subsection, we write $\pi_{iv}(z)$ for the decoder probability of term v in document i under whichever of the two fitted VAE variants is being assessed, evaluated at latent vector z. For every $v\in U_{i}$, a binary completion is sampled from this probability:

(3.23) $${\tilde{x}}_{iv}^{\left(r\right)}∼\mathrm{Bernoulli}\;\left(\pi_{iv}(z_{i}^{\left(r\right)})\right).$$

For $v\in O_{i}$, the revealed entry is fixed at ${\tilde{x}}_{iv}^{\left(r\right)}=x_{iv}=1$ and is excluded from the reported sparsity statistics. The same simulation seed is used for both models.

The observed and simulated numbers of positive terms among the unobserved coordinates in document i are:

(3.24) $$T_{i}^{\mathrm{obs}}=\sum_{v\in U_{i}}x_{iv}\;\text{and}\;T_{i}^{\left(r\right)}=\sum_{v\in U_{i}}{\tilde{x}}_{iv}^{\left(r\right)}.$$

For vocabulary term v, let $N_{v}$ denote its unobserved document count, which is the number of eligible documents in which coordinate v is unobserved. Let $F_{v}^{\mathrm{obs}}$ and $F_{v}^{\left(r\right)}$ denote the observed and simulated document counts for term v among the unobserved coordinates. These quantities are defined by:

(3.25) $$\begin{array}{rl} N_{v} & =\sum_{i\in I}I\{v\in U_{i}\},\\ F_{v}^{\mathrm{obs}} & =\sum_{i\in I}I\{v\in U_{i}\}x_{iv}\\ \text{and }F_{v}^{\left(r\right)} & =\sum_{i\in I}I\{v\in U_{i}\}{\tilde{x}}_{iv}^{\left(r\right)}. \end{array}$$

Positive term counts for documents and document counts for terms are compared with pointwise 90% and 95% Monte Carlo posterior predictive intervals. For terms with $N_{v}>0$, the mean absolute error (MAE) for term rates compares $F_{v}^{\mathrm{obs}}/N_{v}$ with the mean simulated rate across draws. The symbol $\rho_{S}$ denotes Spearman rank correlation between the observed document counts and the mean simulated counts across draws. Appendix C reports the primary 95% summaries (with the full 90% and 95% results generated by the evaluation pipeline available in the project repository).

This design is an approximate conditional posterior predictive check and not a formal goodness of fit test. The mask deliberately holds out positive terms and therefore depends on the observed outcomes rather than representing natural missingness. The intervals integrate latent and Bernoulli sampling uncertainty conditional on fitted weights, the fixed split and the fixed mask. They do not include training, parameter, split or mask uncertainty.

## 4. Results

### 4.1. Model selection and training behaviour

The selected NB VAE reaches its best final fit validation score at epoch 84 with validation completion PPL 557.00. These quantities are used only for model selection, and all performance comparisons below are reported on the test split reserved for final evaluation using the same test documents and completion masks across models.

Figure 3 shows that the training objective with KL warm-up falls quickly in the early epochs and then decreases more gradually, while validation completion PPL reaches its minimum before early stopping. The solid curve represents the mean training objective defined by $J_{e}$ in equation (3.10) (although the figure labels it ‘train negative ELBO’, it equals the negative ELBO only from epoch 30 onward, when $\beta_{e}=1$). No separate unweighted negative ELBO curve is shown during the warm-up period. The selected checkpoint is tied to the prespecified validation completion criterion rather than simply to the final epoch.

**Figure 3.**
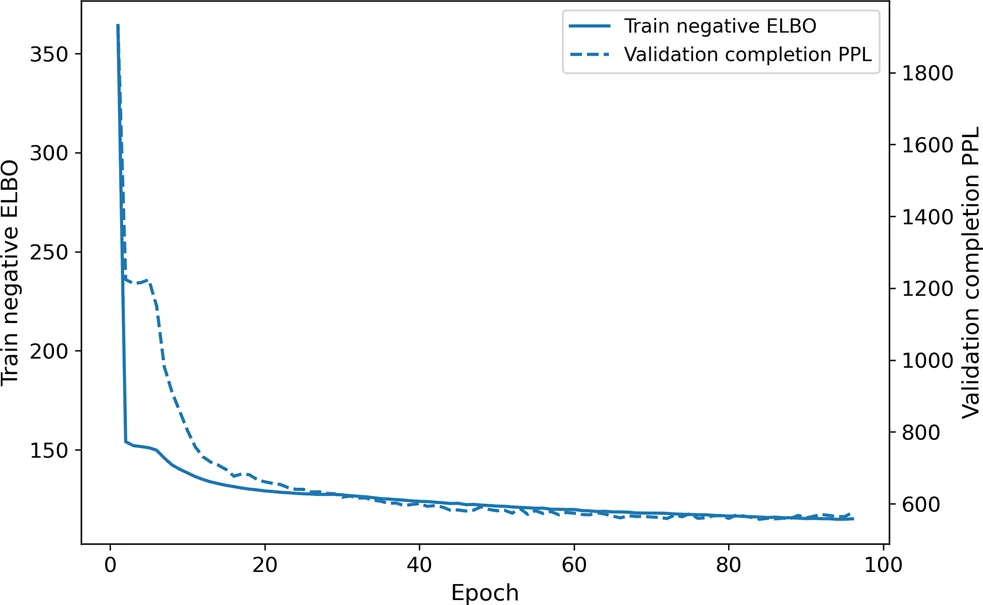
Training dynamics for the final NB VAE fit. The solid curve labelled ‘train negative ELBO’ is the training objective with KL warm-up and equals the negative ELBO from epoch 30 onward. Validation completion PPL reaches its minimum at epoch 84, before final test reporting, while the training objective continues to decrease.

### 4.2. Document completion

Table 2 and figure 1 report document completion performance on the test split reserved for final evaluation. The NB VAE obtains P@5 of 0.351 and nDCG@5 of 0.384. The direct Bernoulli VAE has modestly higher point estimates with P@5 of 0.357 and nDCG@5 of 0.391. Because the evaluation uses one fixed mask, these small differences are descriptive rather than evidence of a general decoder advantage. That said, the negative binomial mapping provides a formal route from a count formulation to term presence but does not improve ranked completion over the direct Bernoulli decoder.

The strongest top-ranked method is the TF-IDF nearest neighbour baseline with P@5 of 0.551 and nDCG@5 of 0.584. This finding clarifies the role of the VAE. When the sole objective is to recover a small number of held out positive terms near the top of a list, lexical nearest neighbour search is a strong baseline for these abstracts. The VAE provides a different output when the task requires a probability for every vocabulary term and a compact representation from the same probabilistic model. The PPL column of table 2 also shows that top-ranked retrieval and the allocation of probability over the full candidate vocabulary are not interchangeable.

Figure 2 extends the comparison beyond the tabulated cut-offs. TF-IDF nearest neighbours retain a substantial recall advantage through cut-off 50, so the bar chart result is not specific to the selected values of k. The nDCG curves provide a complementary view that accounts for rank. The direct Bernoulli and NB VAE curves remain close to the PatentSBERTa and PaECTER curves and above the unigram and ProdLDA curves. Thus, the VAE is not the strongest lexical completion method, but the rankings it provides for individual documents are more informative than global popularity and more competitive than the neural topic model baseline.

### 4.3. Calibration under extreme sparsity

Calibration results are shown in table 3 and figures 4 and 5. On unobserved test document term pairs, the NB VAE has the lowest ECE of 0.001549 and a Brier score of 0.003397. The direct Bernoulli VAE has ECE 0.002167 and Brier score 0.003430, whereas TF-IDF nearest neighbours have the lowest Brier score, 0.002876. The two metrics therefore select different best point estimates. These small values primarily describe the sparse, low-probability region and do not imply that the fitted models reproduce all higher level aspects of the conditional distribution.

**Figure 4.**
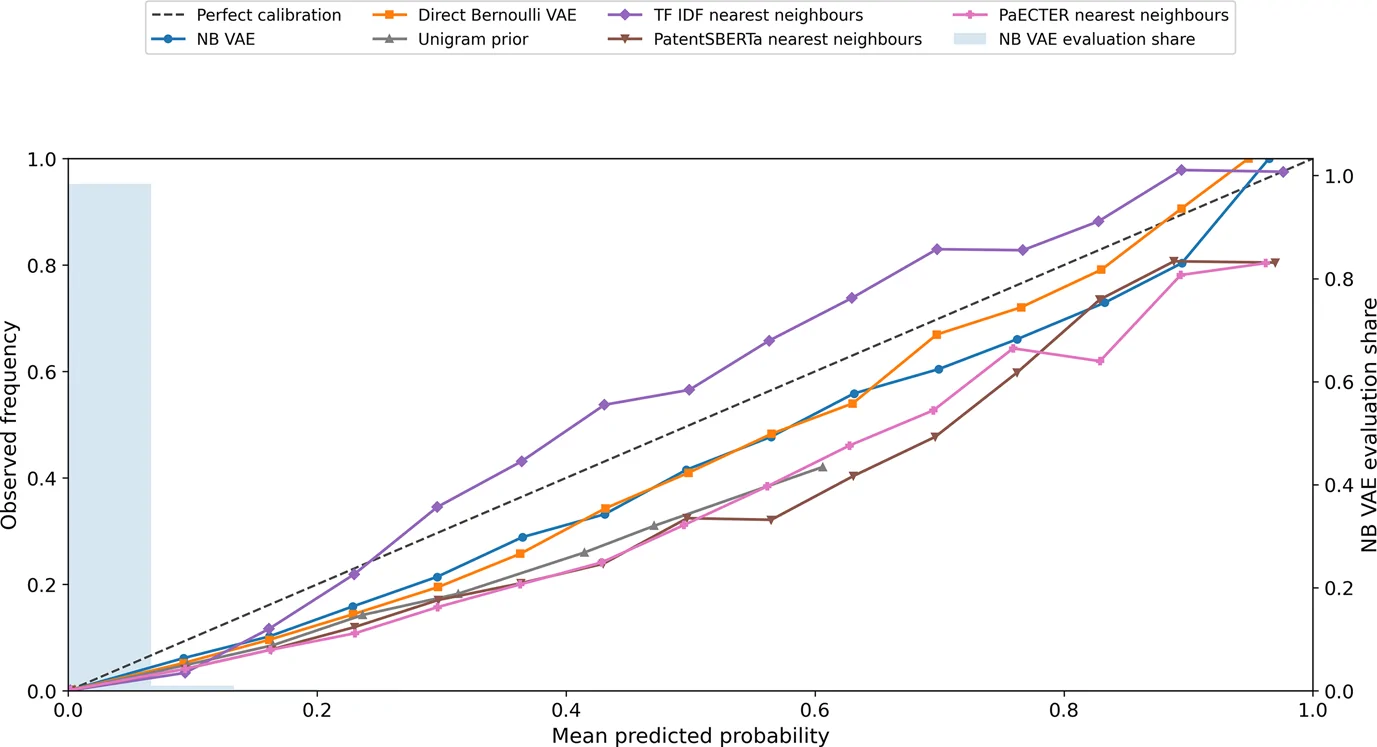
Reliability curves with the NB VAE evaluation share show that aggregate calibration metrics are dominated by probabilities near zero. Points with higher probabilities represent much smaller evaluation shares and should be interpreted accordingly.

**Figure 5.**
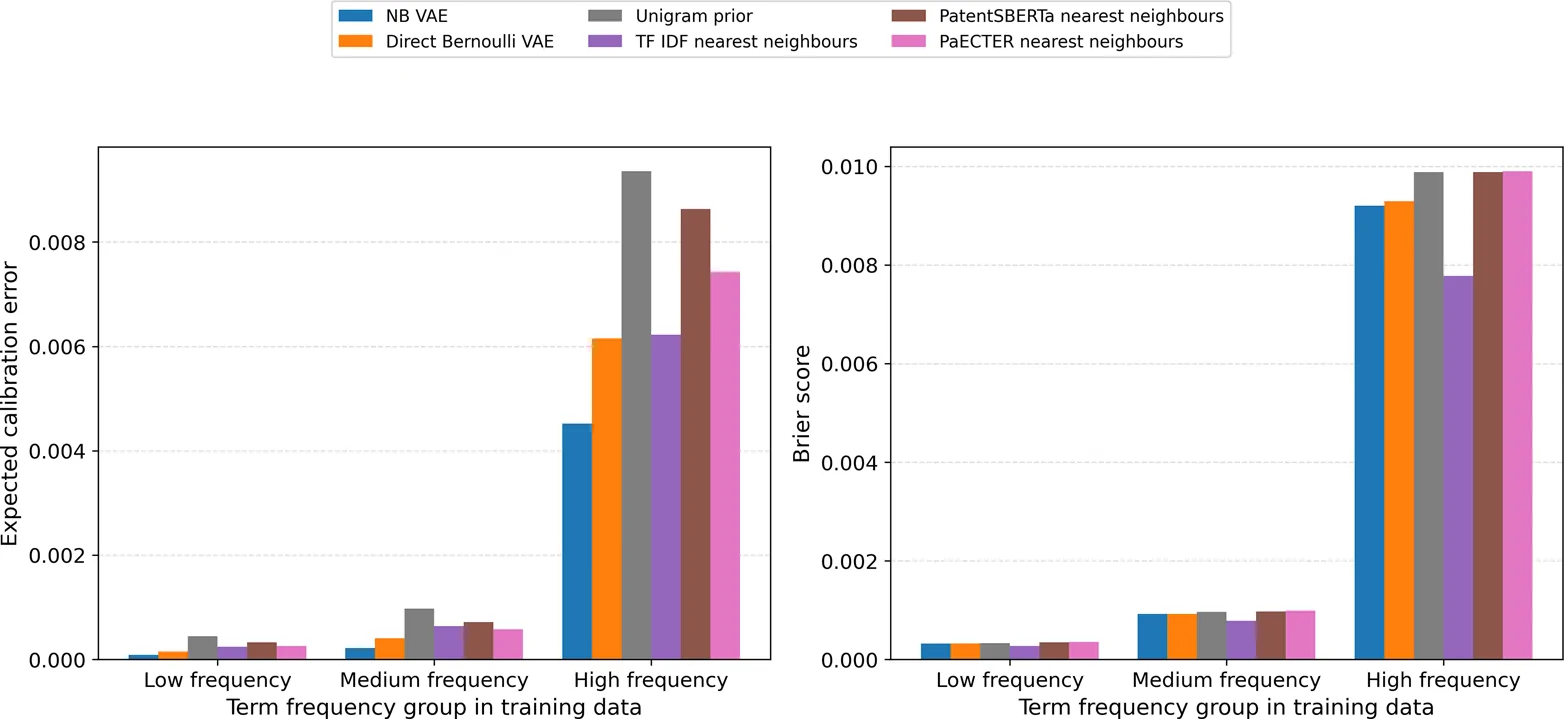
Calibration stratified by training document frequency tertiles. The NB VAE has the lowest ECE in each stratum, while TF-IDF nearest neighbours have the lowest Brier score in each stratum. Absolute errors are largest for common terms.

**Table 3.** Calibration results on unobserved test document term pairs. ECE uses weights based on the number of pairs in each of 15 bins of equal width, so values are driven mainly by the sparse low-probability region. Lower values are better, and the best value in each column is shown in bold. ProdLDA is not shown because its softmax scores form a categorical vocabulary distribution rather than Bernoulli term presence probabilities.

model	ECE	Brier
NB VAE	**0.001549**	0.003397
direct Bernoulli VAE	0.002167	0.003430
unigram prior	0.003506	0.003636
TF-IDF nearest neighbours	0.002187	**0.002876**
PatentSBERTa nearest neighbours	0.003139	0.003644
PaECTER nearest neighbours	0.002682	0.003657

The reliability curves show why scalar calibration values must be interpreted carefully. Nearly all evaluated document term pairs fall in the lowest probability bin, so ECE and Brier score are dominated by assigning small probabilities to absent terms. Bins with higher probabilities contain few pairs, and their deviations are therefore estimated from much less information. The result supports cautious interpretation of the probabilities for sparse vocabulary screening. It does not support a claim of uniform or perfect calibration across the probability range.

Figure 5 helps explain the small aggregate errors. Pairs involving low-frequency terms have extremely low positive rates, so assigning small probabilities to their many absences produces small ECE and Brier values. Absolute errors are larger for common terms. The NB VAE has the lowest ECE within every displayed frequency stratum, while TF-IDF nearest neighbours have the lowest Brier score within every stratum. This pattern depends on the metric and supports cautious interpretation for each task rather than a declaration that one probability model is uniformly best. The conditional draws in §4.4 examine whether the pairwise results also reproduce sparsity at the document and term levels.

### 4.4. Conditional posterior predictive sparsity assessment

Appendix C and table 6 report the conditional posterior predictive results for all 1061 eligible test documents. The observed mean number of positive terms among unobserved coordinates is 16.29. The NB VAE produces a predictive mean of 23.89 with a 95% posterior predictive interval from 23.58 to 24.22 for the mean document count in each draw. The direct Bernoulli VAE produces a predictive mean of 26.56 with an interval from 26.22 to 26.89. Their predictive to observed count ratios are 1.467 and 1.631, respectively. Thus, under the fixed test mask, both fitted models generate more positive terms among the unobserved coordinates than are observed with the NB VAE closer to the observed aggregate sparsity.

**Table 4.** Downstream CPC prefix retrieval on the test split reserved for final evaluation. AP is the primary metric because the positive class is imbalanced within the filtered CCUS corpus. Higher values are better. Within each target prefix, the best value in each column is shown in bold. Four decimals distinguish close point estimates.

target prefix	representation	AUROC	AP	P@100
B01D53/04	TF-IDF SVD	0.9023	**0.7953**	**0.9400**
B01D53/04	PaECTER	**0.9176**	0.7945	0.8900
B01D53/04	NB VAE latent mean	0.7932	0.6427	0.8500
B01D53/04	direct Bernoulli VAE latent mean	0.7894	0.6159	0.7800
B01D53/04	ProdLDA topic proportions	0.5227	0.2674	0.2200
B01D53/62	PaECTER	**0.8367**	**0.6522**	**0.7700**
B01D53/62	TF-IDF SVD	0.8139	0.6223	0.7400
B01D53/62	direct Bernoulli VAE latent mean	0.7627	0.5296	0.6900
B01D53/62	NB VAE latent mean	0.7320	0.4920	0.6400
B01D53/62	ProdLDA topic proportions	0.6086	0.3754	0.4500

**Table 5.** Selected neural model hyperparameters. These are design settings rather than performance results, so no best value is designated.

model	K	encoder widths	decoder form	dropout	learning rate	optimizer
NB VAE	20	(256,128)	mirrored MLP	0.20	$1.0\times 10^{-3}$	AdamW
direct Bernoulli VAE	20	(256,128)	mirrored MLP	0.20	$1.0\times 10^{-3}$	AdamW
ProdLDA	20	(256,128)	topic by word matrix	0.20	$1.0\times 10^{-3}$	AdamW

**Table 6.** Conditional posterior predictive sparsity assessment using 1061 eligible test documents and 1000 draws per fitted model. The observed mean is 16.29 for both rows. The predictive interval summarizes the mean document count in each draw. The predictive to observed count ratio compares simulated and observed positive terms among unobserved coordinates. The MAE for term rates is multiplied by $10^{3}$. Bold font identifies the predictive mean closest to 16.29, the ratio closest to one, the lower error, the higher rank correlation or the interval inclusion fraction closest to 0.95. Bold font does not indicate statistical significance.

*document diagnostics*					
		predictive mean		document	document 95%
model	observed mean	(95% interval)	count ratio $Q_{T}$	$\rho_{S}$	inclusion
NB VAE	16.29	**23.89 (23.58, 24.22)**	**1.467**	0.614	**0.678**
direct Bernoulli VAE	16.29	26.56 (26.22, 26.89)	1.631	**0.633**	0.521

*term diagnostics*			
model	term rate error	term $\rho_{S}$	term 95% inclusion
NB VAE	**2.442**	0.735	**0.916**
direct Bernoulli VAE	2.896	**0.746**	0.897

The observed document count lies within its pointwise 95% posterior predictive interval for 67.8% of documents under the NB VAE and 52.1% under the direct Bernoulli VAE. The direction of most misses is the same. Of the misses, 340 of 342 for the NB VAE and all 508 for the direct Bernoulli VAE fall below the predictive lower bound. Nevertheless, the predicted means retain positive rank association with the observations. The Spearman rank correlations at the document level are 0.614 and 0.633, and those at the term level are 0.735 and 0.746, respectively. The NB VAE is therefore closer in absolute sparsity, whereas the direct Bernoulli VAE has slightly higher rank correlations. Neither comparison establishes general decoder superiority because it is conditional on split and mask.

### 4.5. Downstream retrieval of CPC prefixes

The downstream retrieval task tests whether the representations are useful beyond document completion. Table 4 lists test results for B01D53/04 and B01D53/62, whereas figures 6 and 7 summarize AP and precision-recall behaviour. Both figures identify the citation informed PaECTER representation explicitly.

**Figure 6.**
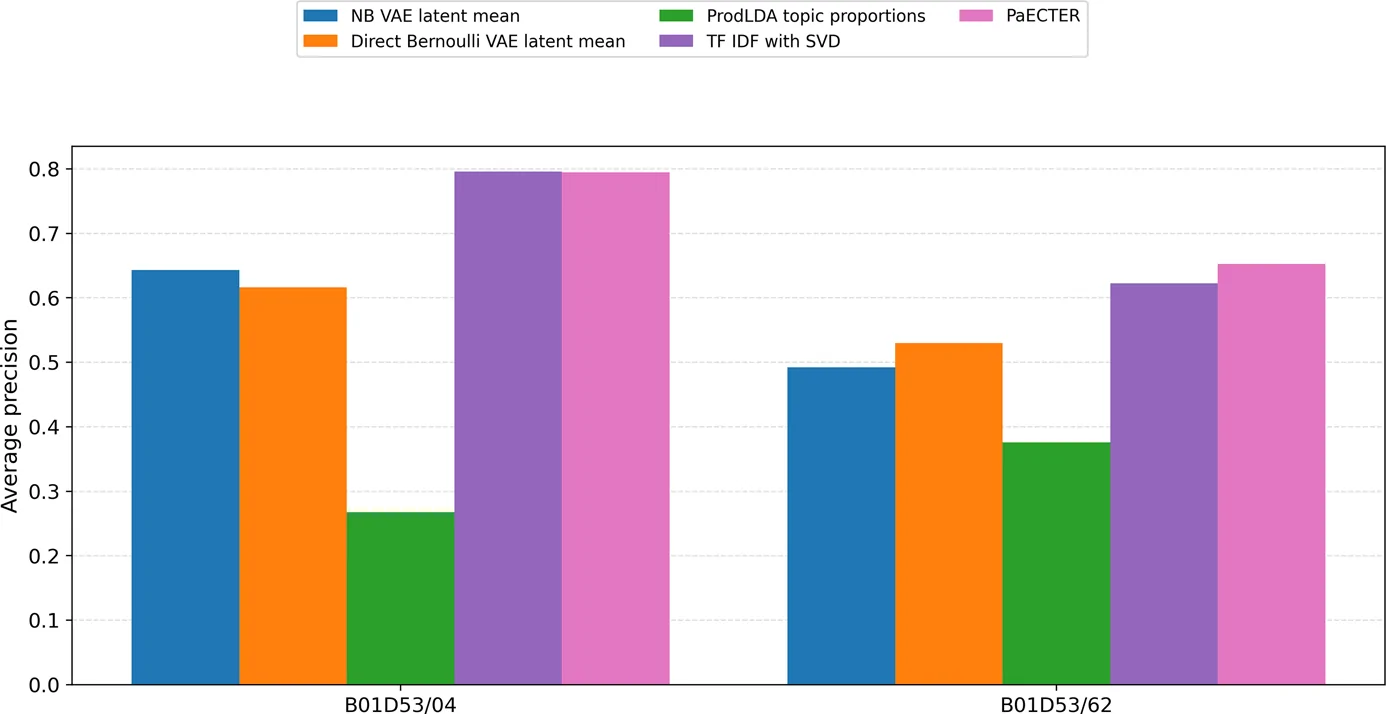
AP for CPC prefix retrieval. TF-IDF after SVD and PaECTER have the highest point estimates, while both VAE latent means retain retrieval signal above ProdLDA.

**Figure 7.**
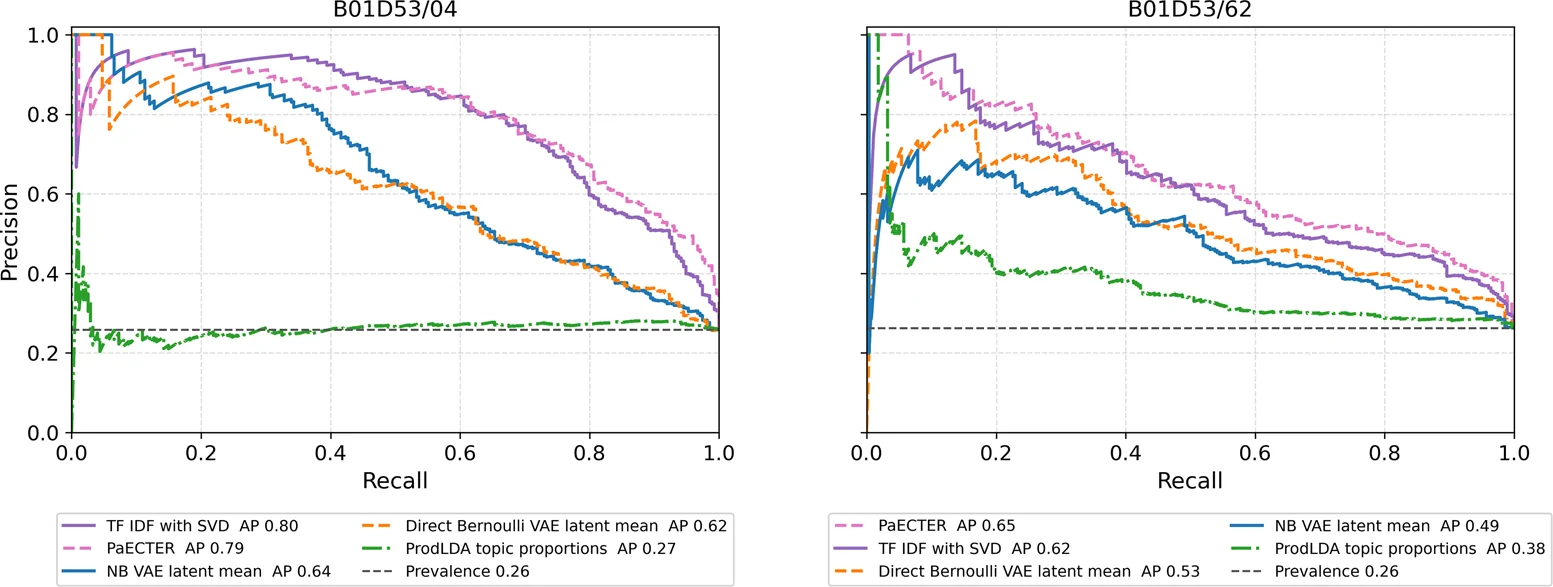
Precision-recall curves for the two CPC prefix targets. TF-IDF after SVD and PaECTER have the highest values over most of the displayed retrieval range, while both VAE representations have higher AP than ProdLDA.

For B01D53/04, PaECTER has the highest AUROC, 0.9176. TF-IDF after SVD has the highest AP, 0.7953, followed numerically by PaECTER at 0.7945. The difference is only 0.0008 and no sampling uncertainty is attached to these point estimates from a single evaluation. The NB VAE and direct Bernoulli VAE latent means have AP 0.6427 and 0.6159, respectively, while ProdLDA is weaker at 0.2674. For B01D53/62, PaECTER has the highest point estimate on all three metrics, including AP 0.6522, compared with 0.6223 for TF-IDF after SVD. The direct Bernoulli VAE reaches AP 0.5296, above the NB VAE at 0.4920 and ProdLDA at 0.3754, but below the lexical and transformer representations. The reversal in the two VAE rankings across target prefixes further cautions against a general claim that either decoder produces the better downstream representation.

Combining the downstream results with completion, calibration and conditional prediction gives a picture that depends on the task. TF-IDF is the strongest when lexical overlap is directly useful, and PaECTER is the strongest or nearly the strongest in downstream CPC prefix retrieval. The VAE is weaker than these baselines for CPC retrieval, but it supplies a different output type. It gives a probability for every vocabulary term that is specific to each document. Reliability diagrams make those probabilities inspectable, while the conditional assessment also reveals their aggregate sparsity mismatch. The output type, rather than retrieval superiority, is the practical distinction of the model in this study.

## 5. Discussion and conclusion

This case study examined whether probabilistic term presence modelling can add useful evidence to CCUS patent analysis. The results support a qualified conclusion. The NB VAE produces a small aggregate pairwise calibration error in the probability region near zero where sparse document term data concentrate, and its latent means retain CPC prefix retrieval signal. However, the direct Bernoulli VAE has modestly higher point estimates for ranked completion, while the relative ordering of the two VAE representations changes across the two CPC prefix targets. TF-IDF nearest neighbours recover held out positive terms more effectively at the top of the ranked list, and TF-IDF after SVD or PaECTER is stronger for downstream CPC prefix retrieval.

This evidence suggests a complementary role rather than a replacement role. CPC labels remain essential because they provide an established administrative structure, while TF-IDF and PaECTER use lexical or pretrained semantic neighbourhoods effectively. A VAE offers a different output as it provides a probability for each vocabulary term that is specific to a document and can be thresholded, ranked, compared across documents and examined with pairwise calibration and conditional predictive checks. Figures 4 and 5 together with §4.4 and appendix C show why both forms of assessment are needed. These probabilities could provide an additional evidence layer for candidate term screening and ranking, although this study does not test whether combining methods improves an operational review process.

The negative binomial to Bernoulli mapping should also be interpreted carefully. It gives a coherent route from a latent count formulation to term presence probabilities with the derivation in appendix A, but the two decoders show different strengths across the diagnostics. The direct Bernoulli VAE is slightly stronger for ranked completion and rank association in the conditional draws. The NB VAE has the lower aggregate ECE and is closer on conditional sparsity levels and interval inclusion. The empirical conclusion is therefore that neither parametrization dominates and that decoder choice remains dependent on the task. The conditional posterior predictive assessment in §4.4 sharpens the calibration interpretation as small pairwise errors in a sparse matrix do not imply that complete draws reproduce sparsity at the document or term level because small probability differences can accumulate over thousands of vocabulary coordinates. Both fitted VAEs generate more positive terms among the unobserved coordinates than observed. The NB VAE is closer on absolute sparsity summaries, whereas the direct Bernoulli VAE retains slightly higher rank correlations. This trade-off supports neither universal decoder dominance nor a claim that either model reproduces the full conditional distribution of abstract sparsity. The probabilities are better interpreted as inspectable scores for candidate term screening and ranking whose aggregate behaviour also requires validation.

Several limitations define the scope of the study. The corpus is restricted to English abstracts selected by CCUS related CPC prefixes. The document representation is binary, which discards term frequency, claims, descriptions, chemical formulae and phrase structure. The completion task evaluates held out positive terms from abstracts rather than full patent relevance, and the CPC prefix task uses administrative labels as an external reference that is not a perfect measure of technological meaning. All reported comparisons come from one fixed split and one fixed completion mask (and as such the recorded seeds make this experiment reproducible but do not quantify split or mask variability). Dependence within patent families or between near duplicate records was not evaluated and may reduce the effective independence of documents across splits.

The conditional assessment has additional limitations. The fixed scheme uses a target masking rate of 50 per cent for the positive terms and depends on the observed outcomes, so it does not represent naturally missing patent text. Furthermore, the encoder is trained on complete binary vectors but receives a partial vector in which unobserved coordinates are filled with zeros. A held out positive term and a genuinely absent term therefore have the same input value. The predictive intervals condition on fitted weights, the fixed split and the fixed mask, and integrate only latent and Bernoulli sampling variability. They exclude training, parameter, split and mask uncertainty and are pointwise rather than simultaneous. Accordingly, the assessment is an approximate conditional model check and not a formal goodness of fit test or evidence of causal or general predictive validity.

Future work could extend the same evaluation design to full patent claims, tokenization based on phrases or chemical information, multiple independent completion masks, temporal splits, splits that account for patent families and repeated model fits. Another possible extension would be to investigate discrete or masked denoising diffusion models operating on binary term presence vectors and conditioned on revealed positive terms. This would align with the present masking and reconstruction protocol and could test whether iterative denoising captures higher-order term dependencies or multiple plausible completions. Calibration and computational cost would need separate evaluation. Finally, hybrid models that combine term presence probabilities with pretrained patent encoders also justify further investigation because the present results show that the two approaches have different strengths.

## Figures and Tables

**Table 7.** Conditional term diagnostics by training document frequency tertile. The first panel compares terms with zero document count among the unobserved coordinates. The second reports rate error, rank correlation and interval inclusion. Predictive zero terms are shown as the mean across draws and a 95% interval. The MAE for term rates is multiplied by $10^{3}$. Within each stratum, bold font identifies the mean zero term count closest to the observed count, the lower error, the higher rank correlation or the interval inclusion fraction closest to 0.95. Bold font does not indicate statistical significance.

*zero count diagnostics*				
model	stratum	terms	observed zero terms	predictive zero terms (95% interval)
NB VAE	rare	1519	1082	**1013.09 (979, 1047)**
direct Bernoulli VAE	rare	1519	1082	956.67 (920, 988.03)
NB VAE	medium	1454	570	**517.20 (485, 551)**
direct Bernoulli VAE	medium	1454	570	445.85 (415, 479.03)
NB VAE	common	1439	59	**47.83 (35, 61)**
direct Bernoulli VAE	common	1439	59	33.36 (23, 44)
***rate, rank and interval diagnostics***				
**model**	**stratum**	**term rate error**	**$\rho_{S}$**	**95% inclusion**
NB VAE	rare	**0.498**	0.169	**0.982**
direct Bernoulli VAE	rare	0.519	**0.211**	0.987
NB VAE	medium	**0.928**	0.211	**0.974**
direct Bernoulli VAE	medium	0.987	**0.241**	0.979
NB VAE	common	**6.024**	0.818	**0.787**
direct Bernoulli VAE	common	7.334	**0.830**	0.719

**Figure 8.**
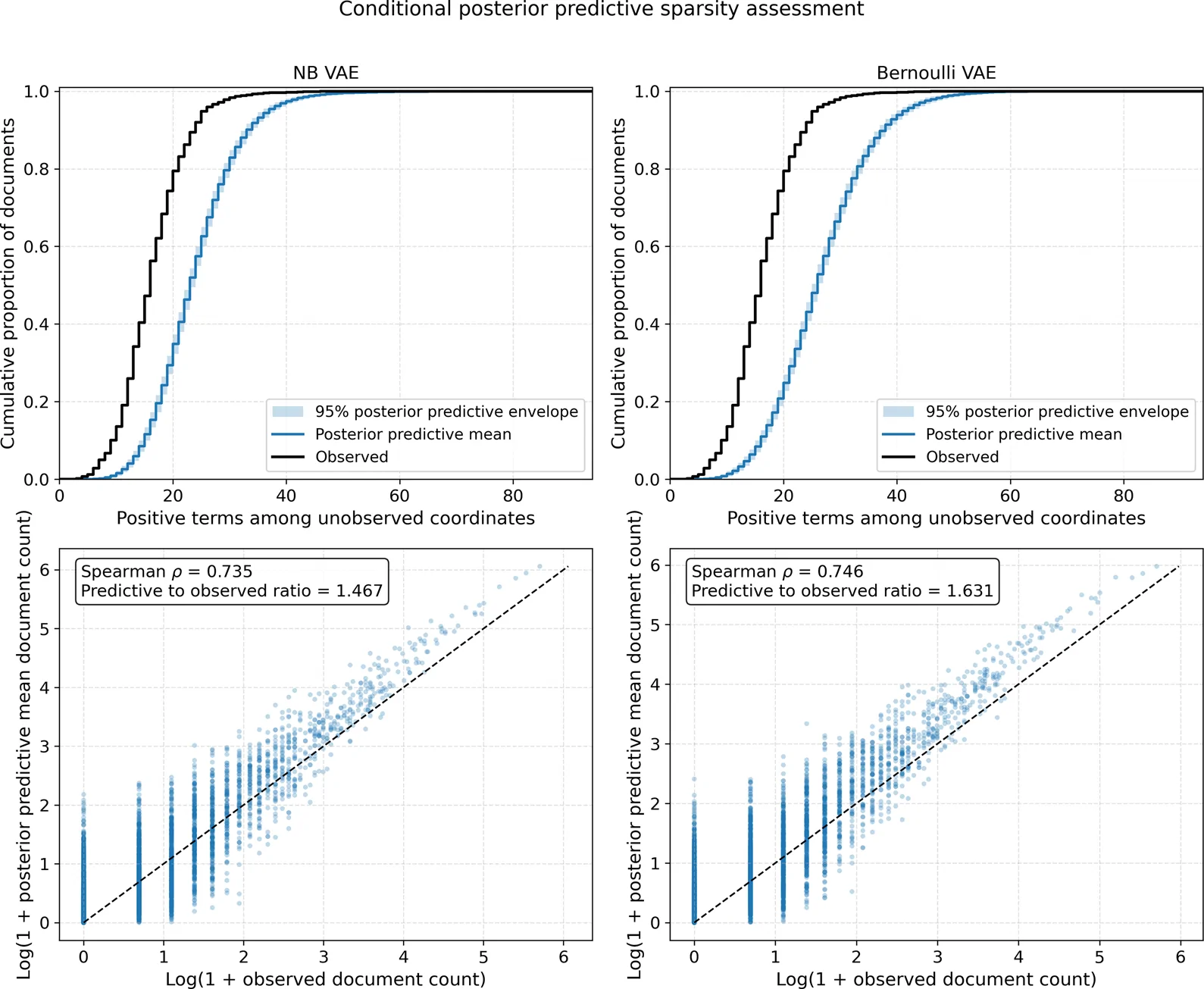
Conditional posterior predictive sparsity assessment. The top panels compare the observed cumulative distribution of positive counts among unobserved coordinates with the mean and pointwise 95% envelope over 1000 simulated cumulative distributions. For each term v, the bottom panels plot $\log(1+F_{v}^{\mathrm{obs}})$ horizontally and $\log(1+{\overset{―}{F}}_{v})$ vertically. The dashed line denotes equality. Spearman rank correlation is denoted by ρ within the figure, and the predictive to observed count ratio is denoted by $Q_{T}$. Revealed positive terms are fixed and excluded from all displayed statistics.

## Data Availability

The source patent records used to construct the study corpus were accessed under confidentiality restrictions and cannot be redistributed. The analytical corpus was derived from these records using the selection and filtering procedures described in §2.1, including restriction to English language records with abstracts and CPC metadata. The derived analytical corpus, complete modelling and evaluation code and generated output are available on GitHub: http://github.com/harismats/VAE_CCUS_Patent_Case_Study and have been archived within the Zenodo repository [27].

## Appendix A. Detailed derivations

This appendix gives the algebra behind the model in §3.1. For document i and vocabulary term v, $x_{iv}\in \{0,1\}$ is the observed term presence indicator, $z_{i}\in R^{K}$ is the latent vector, $\alpha_{iv}>0$ is a negative binomial shape parameter and $\rho_{iv}\in (0,1)$ is the zero probability base in the count formulation. The auxiliary count $m_{iv}$ takes values in {0,1,2,…}, m denotes one possible value and Γ(⋅) denotes the gamma function. The encoder and decoder parameters are ϕ and θ, respectively. The expression $q_{\phi }(z_{i}|x_{i})$ is the encoder density, and $p_{\theta }$ denotes a decoder or joint density as indicated by its arguments.

### A.1. Negative binomial zero probability and Bernoulli presence

With these symbols defined, the negative binomial count formulation has the following probability mass function:

(A.1) $$\mathrm{Pr}(m_{iv}=m|\alpha_{iv},\rho_{iv})=\frac{\Gamma (m+\alpha_{iv})}{\Gamma (\alpha_{iv})\Gamma (m+1)}(1-\rho_{iv}{)}^{m}\rho_{iv}^{\alpha_{iv}}.$$

Set m=0 in the probability mass function:

(A.2) $$\mathrm{Pr}(m_{iv}=0|\alpha_{iv},\rho_{iv})=\frac{\Gamma (\alpha_{iv})}{\Gamma (\alpha_{iv})\Gamma (1)}(1-\rho_{iv}{)}^{0}\rho_{iv}^{\alpha_{iv}}.$$

Use Γ(1)=1 to get:

(A.3) $$\mathrm{Pr}(m_{iv}=0|\alpha_{iv},\rho_{iv})=(1-\rho_{iv}{)}^{0}\rho_{iv}^{\alpha_{iv}},$$

which gives:

(A.4) $$\mathrm{Pr}(m_{iv}=0|\alpha_{iv},\rho_{iv})=\rho_{iv}^{\alpha_{iv}}.$$

The probability of a positive count is one minus the zero probability:

(A.5) $$\mathrm{Pr}(m_{iv}\geq 1|\alpha_{iv},\rho_{iv})=1-\mathrm{Pr}(m_{iv}=0|\alpha_{iv},\rho_{iv}).$$

Substituting the zero probability derived above:

(A.6) $$\mathrm{Pr}(m_{iv}\geq 1|\alpha_{iv},\rho_{iv})=1-\rho_{iv}^{\alpha_{iv}},$$

and define the Bernoulli presence probability $\pi_{iv}$ as:

(A.7) $$\pi_{iv}=1-\rho_{iv}^{\alpha_{iv}}.$$

The binary observation model is therefore given by:

(A.8) $$x_{iv}|z_{i}∼\mathrm{Bernoulli}(\pi_{iv}).$$

### A.2. Stable Bernoulli log likelihood

The Bernoulli likelihood for a single document term pair is given by:

(A.9) $$p(x_{iv}|z_{i})=\pi_{iv}^{x_{iv}}(1-\pi_{iv}{)}^{1-x_{iv}}.$$

Taking logarithms of both sides gives:

(A.10) $$\log p(x_{iv}|z_{i})=\log\left(\pi_{iv}^{x_{iv}}(1-\pi_{iv}{)}^{1-x_{iv}}\right),$$

and using the product rule and power rule for logarithms gives:

(A.11) $$\log p(x_{iv}|z_{i})=x_{iv}\log\pi_{iv}+(1-x_{iv})\log(1-\pi_{iv}).$$

Substituting $\pi_{iv}=1-\rho_{iv}^{\alpha_{iv}}$ into the second logarithm gives:

(A.12) $$\log(1-\pi_{iv})=\log(1-(1-\rho_{iv}^{\alpha_{iv}})).$$

Simplification gives:

(A.13) $$\log(1-\pi_{iv})=\alpha_{iv}\log\rho_{iv},$$

and substituting $\pi_{iv}=1-\rho_{iv}^{\alpha_{iv}}$ into the first logarithm gives:

(A.14) $$\log\pi_{iv}=\log(1-\rho_{iv}^{\alpha_{iv}}).$$

Now, rewriting $\rho_{iv}^{\alpha_{iv}}$ using the exponential function gives:

(A.15) $$\rho_{iv}^{\alpha_{iv}}=\exp(\alpha_{iv}\log\rho_{iv}),$$

and substituting this into $\log\pi_{iv}$ gives:

(A.16) $$\log\pi_{iv}=\log(1-\exp(\alpha_{iv}\log\rho_{iv})).$$

Using expm1⁡(u)=exp⁡(u)−1 avoids the loss of accuracy that can arise from directly subtracting $\exp(\alpha_{iv}\log\rho_{iv})$ from one when $\alpha_{iv}\log\rho_{iv}$ approaches zero:

(A.17) $$\log\pi_{iv}=\log\left[-\mathrm{expm1}(\alpha_{iv}\log\rho_{iv})\right].$$

Combining this expression with the log probability of absence gives the pairwise log likelihood:

(A.18) $$\log p(x_{iv}|z_{i})=x_{iv}\log\left[-\mathrm{expm1}(\alpha_{iv}\log\rho_{iv})\right]+(1-x_{iv})\alpha_{iv}\log\rho_{iv}.$$

Finally, summing over all vocabulary terms gives the document log likelihood:

(A.19) $$\log p_{\theta }(x_{i}|z_{i})=\sum_{v=1}^{\left|V\right|}\left[x_{iv}\log\left[-\mathrm{expm1}(\alpha_{iv}\log\rho_{iv})\right]+(1-x_{iv})\alpha_{iv}\log\rho_{iv}\right].$$

### A.3. Evidence lower bound

Define the joint density $p_{\theta }(x_{i},z_{i})=p_{\theta }(x_{i}|z_{i})p(z_{i})$ and its marginal $p_{\theta }(x_{i})$. The marginal likelihood for document i is given by:

(A.20) $$p_{\theta }(x_{i})=\int p_{\theta }(x_{i},z_{i})\;\text{d}z_{i}.$$

Multiplying and dividing the integrand by the variational density $q_{\phi }(z_{i}|x_{i})$ gives:

(A.21) $$p_{\theta }(x_{i})=\int q_{\phi }(z_{i}|x_{i})\frac{p_{\theta }(x_{i},z_{i})}{q_{\phi }(z_{i}|x_{i})}\;\text{d}z_{i},$$

and recognizing the integral as an expectation under $q_{\phi }(z_{i}|x_{i})$ gives:

(A.22) $$p_{\theta }(x_{i})=E_{q_{\phi }(z_{i}|x_{i})}\left[\frac{p_{\theta }(x_{i},z_{i})}{q_{\phi }(z_{i}|x_{i})}\right].$$

Taking logarithms:

(A.23) $$\log p_{\theta }(x_{i})=\log E_{q_{\phi }(z_{i}|x_{i})}\left[\frac{p_{\theta }(x_{i},z_{i})}{q_{\phi }(z_{i}|x_{i})}\right],$$

and applying Jensen’s inequality to the concave logarithm gives:

(A.24) $$\log p_{\theta }(x_{i})\geq E_{q_{\phi }(z_{i}|x_{i})}\left[\log\frac{p_{\theta }(x_{i},z_{i})}{q_{\phi }(z_{i}|x_{i})}\right].$$

For compactness below, $E_{q_{\phi }}[\cdot ]$ denotes expectation under $q_{\phi }(z_{i}|x_{i})$. Using the quotient rule inside the logarithm gives:

(A.25) $$\log p_{\theta }(x_{i})\geq E_{q_{\phi }}\left[\log p_{\theta }(x_{i},z_{i})-\log q_{\phi }(z_{i}|x_{i})\right].$$

Factoring the joint distribution as $p_{\theta }(x_{i},z_{i})=p_{\theta }(x_{i}|z_{i})p(z_{i})$ gives:

(A.26) $$\log p_{\theta }(x_{i})\geq E_{q_{\phi }}\left[\log p_{\theta }(x_{i}|z_{i})+\log p(z_{i})-\log q_{\phi }(z_{i}|x_{i})\right].$$

Separating the expectation into the likelihood term and the difference between the prior and posterior log densities gives:

(A.27) $$\log p_{\theta }(x_{i})\geq E_{q_{\phi }}[\log p_{\theta }(x_{i}|z_{i})]+E_{q_{\phi }}[\log p(z_{i})-\log q_{\phi }(z_{i}|x_{i})],$$

and using the definition of KL divergence gives:

(A.28) $$D_{\mathrm{KL}}(q_{\phi }(z_{i}|x_{i})\|p(z_{i}))=E_{q_{\phi }}[\log q_{\phi }(z_{i}|x_{i})-\log p(z_{i})].$$

Define the ELBO for document i as $L_{\mathrm{ELBO}}(x_{i})$. The preceding inequality gives:

(A.29) $$L_{\mathrm{ELBO}}(x_{i})=E_{q_{\phi }}[\log p_{\theta }(x_{i}|z_{i})]-D_{\mathrm{KL}}(q_{\phi }(z_{i}|x_{i})\|p(z_{i})).$$

Let e be the training epoch and $\beta_{e}$ the warm-up coefficient defined in §3.1. The objective at epoch e is $J_{e}(x_{i})$. The training loss is the negative reconstruction term plus the weighted divergence:

(A.30) $$J_{e}(x_{i})=-E_{q_{\phi }}[\log p_{\theta }(x_{i}|z_{i})]+\beta_{e}D_{\mathrm{KL}}(q_{\phi }(z_{i}|x_{i})\|p(z_{i})).$$

### A.4. Diagonal Gaussian KL divergence

For this derivation, abbreviate $q_{\phi }(z_{i}|x_{i})$ as q and $p(z_{i})$ as p. Let $\mu_{q}$ and $\mu_{p}$ be their mean vectors, and let $\Sigma_{q}$ and $\Sigma_{p}$ be their covariance matrices. The superscript ⊤ denotes transpose, the superscript −1 denotes a matrix inverse, tr(⋅) denotes a matrix trace and det(⋅) denotes a determinant. With latent dimension K, the multivariate Gaussian KL divergence is given by:

(A.31) $$D_{\mathrm{KL}}(q\|p)=\frac{1}{2}\left(\mathrm{tr}(\Sigma_{p}^{-1}\Sigma_{q})+(\mu_{p}-\mu_{q}{)}^{⊤}\Sigma_{p}^{-1}(\mu_{p}-\mu_{q})-K+\log\frac{\det\;\Sigma_{p}}{\det\;\Sigma_{q}}\right).$$

Here, $q=N(\mu_{i},\mathrm{diag}(\sigma_{i}^{2}))$ and $p=N(0,I_{K})$. Substituting $\mu_{q}=\mu_{i}$, $\mu_{p}=0$, $\Sigma_{q}=\mathrm{diag}(\sigma_{i}^{2})$ and $\Sigma_{p}=I_{K}$ gives:

(A.32) $$D_{\mathrm{KL}}(q\|p)=\frac{1}{2}\left(\mathrm{tr}(I_{K}^{-1}\mathrm{diag}(\sigma_{i}^{2}))+(-\mu_{i}{)}^{⊤}I_{K}^{-1}(-\mu_{i})-K+\log\frac{\det\;I_{K}}{\det\;\mathrm{diag}(\sigma_{i}^{2})}\right),$$

and using $I_{K}^{-1}=I_{K}$ gives:

(A.33) $$D_{\mathrm{KL}}(q\|p)=\frac{1}{2}\left(\mathrm{tr}(\mathrm{diag}(\sigma_{i}^{2}))+(-\mu_{i}{)}^{⊤}(-\mu_{i})-K+\log\frac{\det\;I_{K}}{\det\;\mathrm{diag}(\sigma_{i}^{2})}\right).$$

Let k∈{1,…,K} index a latent coordinate, and let $\mu_{ik}$ and $\sigma_{ik}$ be the kth entries of $\mu_{i}$ and $\sigma_{i}$, respectively. Evaluating the trace of the diagonal covariance gives:

(A.34) $$\mathrm{tr}(\mathrm{diag}(\sigma_{i}^{2}))=\sum_{k=1}^{K}\sigma_{ik}^{2}.$$

Furthermore, evaluating the quadratic term gives:

(A.35) $$(-\mu_{i}{)}^{⊤}(-\mu_{i})=\mu_{i}^{⊤}\mu_{i}=\sum_{k=1}^{K}\mu_{ik}^{2},$$

then, evaluating the determinant of the identity matrix gives:

(A.36) $$\det I_{K}=1,$$

and evaluating the determinant of the diagonal covariance matrix gives:

(A.37) $$\det\mathrm{diag}(\sigma_{i}^{2})=\prod_{k=1}^{K}\sigma_{ik}^{2}.$$

Substituting these four results into the KL expression gives:

(A.38) $$D_{\mathrm{KL}}(q\|p)=\frac{1}{2}\left(\sum_{k=1}^{K}\sigma_{ik}^{2}+\sum_{k=1}^{K}\mu_{ik}^{2}-K+\log\frac{1}{\prod_{k=1}^{K}\sigma_{ik}^{2}}\right).$$

Using log⁡(1/a)=−log⁡a gives:

(A.39) $$D_{\mathrm{KL}}(q\|p)=\frac{1}{2}\left(\sum_{k=1}^{K}\sigma_{ik}^{2}+\sum_{k=1}^{K}\mu_{ik}^{2}-K-\log\prod_{k=1}^{K}\sigma_{ik}^{2}\right).$$

For positive values $a_{1},\ldots ,a_{K}$, using $\log\prod_{k=1}^{K}a_{k}=\sum_{k=1}^{K}\log a_{k}$ gives:

(A.40) $$D_{\mathrm{KL}}(q\|p)=\frac{1}{2}\left(\sum_{k=1}^{K}\sigma_{ik}^{2}+\sum_{k=1}^{K}\mu_{ik}^{2}-K-\sum_{k=1}^{K}\log\sigma_{ik}^{2}\right),$$

and rewriting K as a sum of ones and collecting terms gives:

(A.41) $$D_{\mathrm{KL}}(q\|p)=\frac{1}{2}\sum_{k=1}^{K}\left(\mu_{ik}^{2}+\sigma_{ik}^{2}-\log\sigma_{ik}^{2}-1\right).$$

## Appendix B. Reproducibility details

This appendix records the computational details. The corpus is divided once into training, validation and test shares of 80 per cent, 10 per cent and 10 per cent, respectively, using seed 10 and joint stratification on the B01D53/04 and B01D53/62 indicators. The binary count vectorizer is fitted only on the training split, and its fitted vocabulary is then applied unchanged to validation and test abstracts. The validation and test masks use seeds 11 and 12, respectively. Every model is evaluated on the same mask within a task.

The three neural models are trained for at most 150 epochs with early stopping patience 12, batch size 64, gradient clipping at 50, weight decay 0.01 and a 30 epoch linear warm-up of the KL term. The optimizer is AdamW. Python, NumPy and PyTorch random states and the data loader generator are seeded, and an NVIDIA A100 graphics processing unit with 40 gigabytes of memory was used.

The NB VAE validation grid considers latent dimensions 20 and 50, hidden layer widths (256,128), (384,128) and (384,64), dropout rates 0.20 and 0.35 and learning rates from $5\times 10^{-4}$ to $10^{-3}$. The selected configuration is refitted under the matched final training protocol. In that final fit, the NB VAE reaches minimum validation completion PPL 556.998 at epoch 84. The direct Bernoulli VAE reaches 557.451 at epoch 98, and ProdLDA reaches 997.155 at epoch 148. These are validation diagnostics, not test results.

In table 5, K denotes the latent dimension for either VAE and the number of topics for ProdLDA. A multi-layer perceptron (MLP) is the feed-forward encoder or decoder network used by the VAE variants.

The same selected encoder capacity is used for the direct Bernoulli VAE and ProdLDA so that the comparison retains the main training conditions. Hidden layers use the rectified linear unit activation, and the mirrored MLP decoder expands through widths (128,256). The NB VAE decoder has two separate linear output heads, each of width |V|. One produces $a_{iv}$ which determines $\alpha_{iv}$, and the other produces $b_{iv}$ which determines $\rho_{iv}$. The direct Bernoulli VAE instead uses one linear output head of width |V|. The NB VAE uses count logit clipping constant $a_{\max}=10$. The direct Bernoulli VAE and ProdLDA do not use that link.

Additional numerical safeguards are applied when evaluating the decoder links. Let $\varepsilon_{\alpha }=10^{-6}$ and $\varepsilon_{\pi }=10^{-7}$. The implemented shape is $\alpha_{iv}=\exp\{\mathrm{clip}(a_{iv},-a_{\max},a_{\max})\}+\varepsilon_{\alpha }$. Let $\ell_{iv}=\min\{\alpha_{iv}\log\rho_{iv},-\varepsilon_{\pi }\}$ be the capped log zero probability and let expm1⁡(u)=exp⁡(u)−1. For the NB VAE loss, the log probability of a positive observation is evaluated as $\log[-\mathrm{expm1}(\ell_{iv})]$, while the log probability of a zero observation is $\ell_{iv}$. For prediction and conditional simulation, the returned NB VAE presence probability is $\pi_{iv}^{\mathrm{impl}}=\mathrm{clip}(-\mathrm{expm1}(\ell_{iv}),\varepsilon_{\pi },1-\varepsilon_{\pi })$. The direct Bernoulli VAE uses binary cross entropy with logits during training and clips its sigmoid probability to the same interval for prediction and conditional simulation. These safeguards avoid undefined log probabilities and keep the probabilities used for prediction and simulation away from zero and one while retaining the decoder links defined in §3.1.

Table 6 shows a distinction between ordering and absolute level. The direct Bernoulli VAE has slightly higher rank correlations at the document and term levels, whereas the NB VAE is closer to the observed sparsity level on the predictive mean, count ratio, term rate error and interval inclusion summaries. The observed mask contains 1711 terms with zero document count among the unobserved coordinates. Posterior predictive draws contain a mean of 1578 such terms under the NB VAE with a 95% interval from 1526 to 1626, and the corresponding mean is 1436 under the direct Bernoulli VAE with an interval from 1386 to 1485. The observed value lies above both intervals, so both fitted models generate non-zero occurrences for more terms than observed.

The nearest neighbour baselines use 30 cosine neighbours, and TF-IDF scores are non-negative averages of neighbour term presence vectors weighted by cosine similarity truncated at zero with a $10^{-6}$ offset before normalization. For completion, the TF-IDF query vector and the constructed abstracts supplied to the patent transformer models contain only revealed positive terms. PatentSBERTa and PaECTER are evaluated separately. For downstream CPC prefix retrieval, full abstract representations are used. TF-IDF is reduced to 50 SVD dimensions, and all representations are evaluated with logistic regression using balanced class weights at most 2000 iterations and seed 10.

## Appendix C. Conditional posterior predictive assessment

This appendix expands the design in §3.4 and its findings. Recall the eligible test document set $I=\{i||H_{i}|>0\}$ and let $N_{\mathrm{doc}}=|I|=1061$. The symbols R=1000, $T_{i}^{\mathrm{obs}}$, $T_{i}^{\left(r\right)}$, $N_{v}$, $F_{v}^{\mathrm{obs}}$ and $F_{v}^{\left(r\right)}$ are defined in §3.4.

Define the observed mean document count ${\overset{―}{T}}^{\mathrm{obs}}$ and the mean for draw r, ${\overset{―}{T}}^{\left(r\right)}$ by:

(C.1) $${\overset{―}{T}}^{\mathrm{obs}}=\frac{1}{N_{\mathrm{doc}}}\sum_{i\in I}T_{i}^{\mathrm{obs}}\;\text{and}\;{\overset{―}{T}}^{\left(r\right)}=\frac{1}{N_{\mathrm{doc}}}\sum_{i\in I}T_{i}^{\left(r\right)}.$$

The reported interval beside the predictive mean is formed from the 2.5th and 97.5th percentiles of $\left\{{\overset{―}{T}}^{\left(r\right)}|r=1,\ldots ,R\right\}$. Define the predictive to observed count ratio $Q_{T}$ as:

(C.2) $$Q_{T}=\frac{R^{-1}\sum_{r=1}^{R}\sum_{i\in I}T_{i}^{\left(r\right)}}{\sum_{i\in I}T_{i}^{\mathrm{obs}}}.$$

For each term v, define its mean simulated document count across draws as ${\overset{―}{F}}_{v}=R^{-1}\sum_{r=1}^{R}F_{v}^{\left(r\right)}$. Let $V_{+}=\{v\in V|N_{v}>0\}$ be the set of terms with a positive unobserved document count. The MAE for term rates, denoted ${\mathrm{MAE}}_{\mathrm{rate}}$ is:

(C.3) $${\mathrm{MAE}}_{\mathrm{rate}}=\frac{1}{\left|V_{+}\right|}\sum_{v\in V_{+}}\left|\frac{{\overset{―}{F}}_{v}}{N_{v}}-\frac{F_{v}^{\mathrm{obs}}}{N_{v}}\right|.$$

Tables 6 and 7 list $10^{3}\times {\mathrm{MAE}}_{\mathrm{rate}}$. The symbol $\rho_{S}$ denotes Spearman rank correlation. An interval inclusion fraction is the proportion of observed document or term statistics inside their corresponding pointwise posterior predictive intervals from the 2.5th to the 97.5th percentile. It is a descriptive inclusion measure and not a frequentist coverage probability.

Table 7 qualifies the high overall term inclusion fractions. The absolute term rate error is the largest among common terms for both models. Conversely, high inclusion for rare and medium-frequency terms partly reflects their discrete frequencies with many zero counts and should not by itself be interpreted as accurate reproduction of individual low-frequency terms. Across all three strata, the NB VAE is closer on absolute sparsity and rate error, whereas the direct Bernoulli VAE has the higher rank correlation.

Figure 8 makes both parts of the result visible. Predictive document count distributions are shifted towards larger counts, yet the mean simulated document counts for terms retain a positive monotonic association with their observed counterparts. The assessment remains conditional on the fitted weights, fixed split and fixed mask that depends on the observed outcomes, as detailed in §3.4.
